# Effects of High-Resolution CT Changes on Prognosis Predictability in Acute Respiratory Distress Syndrome with Diffuse Alveolar Damage

**DOI:** 10.3390/jcm11092458

**Published:** 2022-04-27

**Authors:** Ching-Ying Huang, Patricia Wanping Wu, Yon-Cheong Wong, Kuo-Chin Kao, Chung-Chi Huang

**Affiliations:** 1 Department of Medical Imaging and Intervention, Linkou Chang Gung Memorial Hospital, Taoyuan 33353, Taiwan; noctis.huang@gmail.com (C.-Y.H.); pwwu@cgmh.org.tw (P.W.W.); ycwong@cgmh.org.tw (Y.-C.W.); 2 Department of Respiratory Therapy, College of Medicine, Chang Gung University, Taoyuan 33353, Taiwan; kck0502@cgmh.org.tw; 3 Department of Pulmonary and Critical Care Medicine, Linkou Chang Gung Memorial Hospital, Taoyuan 33353, Taiwan; 4 Department of Respiratory Therapy, Linkou Chang Gung Memorial Hospital, Taoyuan 33353, Taiwan

**Keywords:** acute respiratory distress syndrome, diffuse alveolar damage, high-resolution computed tomography, traction bronchiectasis, mechanical power

## Abstract

Diffuse alveolar damage (DAD) is the pathological hallmark of acute respiratory distress syndrome (ARDS). DAD is independently correlated with higher mortality compared with the absence of DAD. Traction bronchiectasis in areas of ground-glass opacity or consolidation is associated with the late fibroproliferative or fibrotic phase of DAD. This study examined whether the 60-day mortality related to DAD could be predicted using high-resolution computed tomography (HRCT) findings and HRCT scores. A total of 34 patients with DAD who received HRCT within 7 days of ARDS diagnosis were enrolled; they were divided into a 60-day survival group and a nonsurvival group, with 17 patients in each group. Univariate and multivariate binary regression analyses and the receiver operating characteristic curve revealed that only the total percentage of the area with traction bronchiectasis or bronchiolectasis was an independent predictor of 60-day mortality (odds ratio, 1.067; 95% confidence interval (CI), 1.011–1.126) and had favorable predictive performance (area under the curve (AUC): 0.784; 95% CI, 0.621–0.946; cutoff, 21.7). Physiological variables, including age, days from ARDS to HRCT, the sequential organ failure assessment (SOFA) score, the PaO_2_/fraction of inspired oxygen (FiO_2_) ratio, dynamic driving pressure, and dynamic mechanical power, were not discriminative between 60-day survival and nonsurvival. In conclusion, the extent of fibroproliferation on HRCT in early ARDS, presented as the total percentage of area with bronchiectasis or bronchiolectasis, is an independent positive predictor with a favorable predictive ability for the 60-day mortality of DAD.

## 1. Introduction

Acute respiratory distress syndrome (ARDS) is the most severe form of acute lung injury in the ICU. The LUNG SAFE study revealed that the incidence of ARDS among ICU admissions was 10.4% [1]. ARDS-related mortality was over 40% in two large cohorts and has remained high in the past 10 years [2,3]. Regarding the mortality for different severities, the recent LUNG SAFE study disclosed that hospital mortality was 34.9% for those with mild, 40.3% for those with moderate, and 46.1% for those with severe ARDS [1]. Diffuse alveolar damage (DAD), characterized by interstitial edema, type II cell hyperplasia, acute inflammation, and hyaline membranes, is the pathological hallmark of the acute exudative phase of ARDS [3]. ARDS patients tend to progress to the fibroproliferative phase and then the fibrotic phase if they survive after the initial exudative phase [4]. However, several autopsy [5] and open lung biopsy (OLB) [6,7,8] studies have revealed that only approximately half of patients with ARDS have DAD. Compared with the absence of DAD, DAD is associated with greater disease severity and is an independent risk factor for higher mortality [5,6,7,8]. However, the PREDATOR study concluded that DAD cannot be predicted merely on the basis of clinical parameters and that tissue biopsy is required for the histopathological diagnosis of DAD [9]. OLB, a routine procedure, is complex and presents various risks; therefore, the authors proposed that developing alternate methods, such as biomarkers and lung imaging techniques, for diagnosing DAD is necessary.

High-resolution computed tomography (HRCT) findings have substantial value in ARDS diagnosis [10,11,12,13] and correspond to the pathological phases of DAD [14,15,16]. The absence of traction bronchiectasis or bronchiolectasis in areas of ground-glass opacity (GGO) or consolidation corresponds to the exudative and early proliferative phases of DAD, whereas the presence of traction bronchiectasis or bronchiolectasis in areas of GGO or consolidation is associated with the late proliferative and fibrotic phases of such damage.

Ichikado et al. [17] assigned different weights to HRCT findings, including normal attenuation, GGO, consolidation, traction bronchiectasis, and honeycombing, and established a scoring system to evaluate the severity of fibroproliferative changes in the lung parenchyma. They reported that extensive fibroproliferative changes on HRCT were an independent predictor of poor prognosis in the clinical early stage of ARDS. They also revealed that HRCT scores <230 could predict 28-day survival after ARDS onset (area under the receiver operating characteristic curve (AUROC), 0.808; sensitivity, 73%; specificity, 75%) and were associated with a lower incidence of barotrauma and a greater number of ventilator-free days. They also proposed that in patients with early ARDS, the extent of pulmonary fibroproliferation assessed using HRCT and an HRCT score of >210 could predict an increase in 60-day mortality (AUROC, 0.71; 95% confidence interval (CI), 0.61–0.82; sensitivity, 71%; specificity, 72%), with a greater susceptibility of these patients to multiple organ failure and ventilator dependency and their associated outcomes [18].

The risk of hospital mortality related to cases with DAD has been reported to be approximately two times higher than that related to cases without DAD [6,9]. Because of the poorer prognosis of DAD, this study examined whether changes in and scores of HRCT performed within 7 days of ARDS diagnosis could predict the 60-day mortality of patients with ARDS with pathologically confirmed DAD.

## 2. Materials and Methods

### 2.1. Patient Enrollment

We retrospectively reviewed the medical charts of all ARDS cases that fulfilled the Berlin definition criteria [3] and received lung biopsies at the Linkou Chang Gung Memorial Hospital after 2003. A total of 126 cases with lung biopsies were collected, including 105 patients that received open lung biopsy (OLB) and 21 patients that received cryobiopsy. In most of these cases, lung biopsy was indicated when ARDS was suspected to be noninfectious and the patient did not exhibit clear risk factors. The clinical and radiological manifestations progressed rapidly. The infiltrations, GGO, or consolidation revealed on chest X-ray or HRCT were bilateral, with a relatively symmetrical distribution. Informed consent for OLB or cryobiopsy was obtained from the patients’ families.

**Figure 1:**
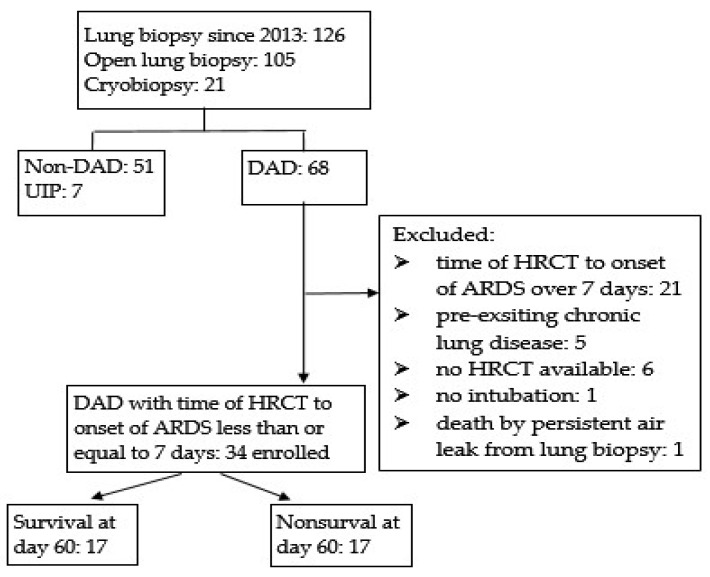
Flow chart for patient enrollment. DAD: diffuse alveolar damage; UIP: usual interstitial pneumonia.

A flowchart for patient enrollment and exclusion is presented in Figure 1. Of the 126 patients with lung biopsies, 68 had a pathological diagnosis of DAD. The remaining 51 did not have DAD; 7 cases with usual interstitial pneumonia were excluded. Of the 68 DAD cases, 21 cases with a time from HRCT to ARDS diagnosis of more than 7 days, 6 cases with no available HRCT data, and 5 cases of chronic lung disease according to the clinical history or previous radiological findings were also excluded. Because this study was retrospective, it was difficult to have HRCT performed at the time of diagnosis of ARDS in every case. For the first week after ARDS onset, the main histopathology is the acute exudative phase, followed by the fibroproliferative and fibrotic phases from the second week onward. We wanted to know whether the ARDS patients had entered into the fibroproliferative or fibrotic phases in the clinical early stage at the time of ARDS diagnosis. If the HRCT was performed over 7 days after ARDS diagnosis, it was reasonable that the HRCT would reveal fibroproliferative changes because of disease progression. Therefore, only DAD patients with HRCT performed within 7 days of ARDS diagnosis were enrolled. Finally, 34 patients with DAD were enrolled and divided into two groups, the 60-day survival group and the nonsurvival group, with 17 patients in each group. In this study, we defined 60-day survival as either (1) patients who remained admitted or (2) patients who were discharged but whose OPD follow-up record could be found over 60 days after admission. The Institutional Review Board (IRB) of Chang Gung Memorial Hospital approved the study protocol (IRB number 202200184B0), and the study was performed in compliance with the tenets of the Declaration of Helsinki. The IRB waived the need for written informed consent because this study was retrospective and had no bearing on modifications to patient management. All personal information in the database were encrypted and deidentified.

The pathological criteria for DAD diagnosis included the presence of hyaline membranes, pulmonary inflammatory infiltrates, intra-alveolar and interstitial edema, alveolar type II cell hyperplasia, interstitial proliferation of fibroblasts and myofibroblasts, and organizing interstitial fibrosis.

### 2.2. Clinical Data

The following clinical data were retrieved from the patients’ medical charts: age, sex, body weight, underlying diseases, ARDS diagnosis, the time from HRCT and lung biopsy to ARDS diagnosis, the time from lung biopsy to HRCT, the duration of mechanical ventilation until ventilator weaning or patient death, the sequential organ failure assessment (SOFA) score, the PaO_2_/FiO_2_ (P/F) ratio, tidal volume, the positive end-expiratory pressure (PEEP) level, peak airway pressure, dynamic driving pressure, dynamic mechanical power (MP), and the severity of ARDS according to the Berlin definition criteria [3]. The clinical data, Coma scale, and SOFA score were recorded at the time of diagnosis of ARDS before sedation and paralysis.

### 2.3. Examination, Assessment, and Scoring of HRCT

Whole-lung volumetric HRCT scans were performed using multidetector-row computed tomography (CT) at full inspiration from the lung apex to the base. All multidetector-row CT scans were obtained with a 2.5 mm section thickness and a table speed per rotation of 15 mm. Abutting CT slices were reconstructed using a high-spatial frequency algorithm. The sections were displayed at 10 mm intervals throughout the chest with the patient in a supine position.

**Figure 2:**
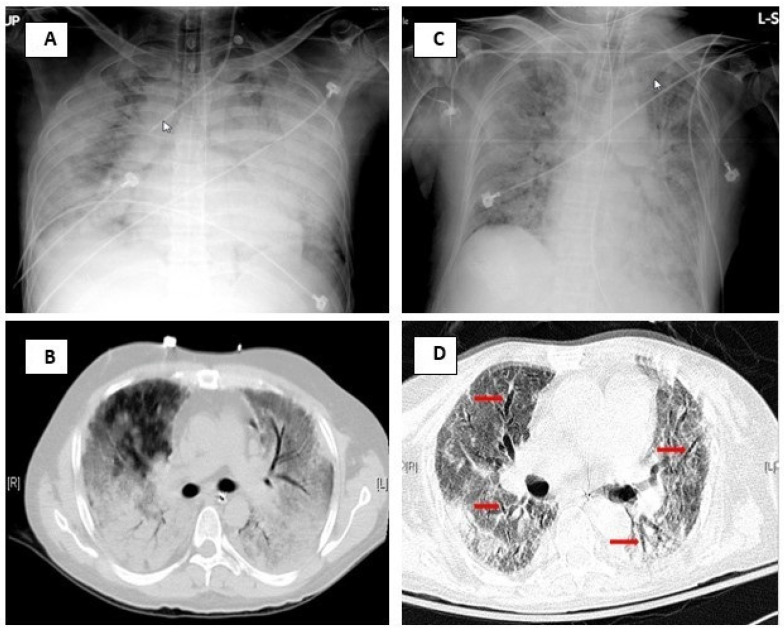
(**A**,**B**) Chest radiography and HRCT scan at the level below carina of a 35-year-old male patient with psoriasis with ARDS caused by methotrexate-induced pneumonitis who survived and was discharged. (**C**,**D**) Chest radiography and HRCT scan at the level below the carina of a 75-year-old female with left parotid gland lymphoepithelial carcinoma with liver and bone metastasis and ARDS caused by pneumonia who expired in ICU. Both chest X-rays (**A**,**C**) show bilateral dense consolidation, more severe in (**A**) the survival case. (**B**) HRCT findings of the survival case show extensive GGO and consolidation with a smooth bronchial wall. (**D**) HRCT findings in the non-survival case show bilateral GGO, reticulation, and prominent traction bronchiectasis (red arrows).

According to the recommendations of the Nomenclature Committee of the Fleischner Society [19], the HRCT abnormalities were defined as follows: GGO—hazy areas denoting increased lung attenuation, but with preservation of bronchial and vascular margins; consolidation—homogeneous increase in pulmonary parenchymal attenuation that obscures the margins of vessels and airway walls; traction bronchiectasis—bronchial dilatation, which is commonly irregular, in association with juxtabronchial opacification that is interpreted as representing retractile pulmonary fibrosis; traction bronchiolectasis—bronchiolar dilatation in association with peribronchiolar opacification that is interpreted as representing retractile pulmonary fibrosis; honeycombing—clustered cystic air spaces (between 0.3 and 1.0 cm in diameter, but occasionally as large as 2.5 cm), which are usually subpleural and characterized by well-defined, often thick, walls. The chest radiography and HRCT scans of a case in the survival group and a case in the nonsurvival group are illustrated in Figure 2.

We adopted the HRCT scoring system proposed by Ichikado et al. to evaluate the severity of parenchyma abnormalities. The scoring system was previously used in the evaluation of HRCT findings of patients with acute interstitial pneumonia (AIP) [16], ARDS [17,18], and idiopathic pulmonary fibrosis (IPF) [20]. The HRCT findings were ranked on a scale of 1–6 on the basis of the classification system, with the scores indicating the following: 1—normal attenuation, 2—GGO, 3—consolidation, 4—GGO with traction bronchiectasis or bronchiolectasis, 5—consolidation with traction bronchiectasis or bronchiolectasis, and 6—honeycombing. The left and right lungs were divided into an upper zone (the area above the level of the carina), a middle zone (the area between the carina and infrapulmonary vein), and a lower zone (the area below the level of the infrapulmonary vein). The extent of each of these six abnormalities was assessed independently in each of the six zones (i.e., the upper, middle, and lower zones of the left and right lungs). The proportion of each HRCT abnormality in the affected lung parenchyma in each zone was assessed through a visual estimation of the percentage (to the nearest 10%). The abnormality score for each zone was calculated by multiplying the percentage area by the ranking score obtained (1–6). The total score for each abnormality was calculated as the average of the scores of the six zones for each patient. The final CT score for each patient was obtained through the summation of the six averaged scores. The HRCT scores were independently evaluated by one pulmonologist and one radiologist. The assessment scores assigned by these two observers were averaged.

Because our study was retrospective, and because data on plateau pressure and static measurements were not available for all cases, the dynamic driving pressure and dynamic MP were applied in this cohort. The dynamic driving pressure was calculated by subtracting the PEEP from the peak inspiratory pressure. The dynamic MP was calculated as follows: 0.098 × respiratory rate × tidal volume × [peak inspiratory pressure—(0.5 × dynamic driving pressure)] [21].

### 2.4. Statistical Analysis

All variables are expressed as means ± standard deviation. The Kolmogorov–Smirnov test was used to verify the normality of the distributions of continuous variables. Differences in the continuous variables between the survival and nonsurvival groups were analyzed using Student’s *t*-test or Mann–Whitney *U* test. Differences in categorical variables between the survival and nonsurvival groups were compared using the chi-square test or Fisher’s exact test. Univariate binary logistic regression model analyses were applied to evaluate the association between the 60-day mortality and the selected variables. The variables significantly associated with the outcome of 60-day mortality were input into the multivariate logistic regression model to assess their independent contribution to the outcome. The ability of the studied variables to predict 60-day mortality was tested using the receiver operating characteristic (ROC) curve. The area under each ROC curve (AUC) was calculated, with a value of <0.5 indicating the inability of the indicator to accurately predict 60-day mortality. The cutoff value for the data was based on the maximum value of Youden’s index (J = sensitivity + specificity − 1). Linear regression and correlation analysis were used to analyze the association of the HRCT scores and total percentage of the area with traction bronchiectasis with ARDS severity (P/F ratio) and lung mechanics (dynamic MP). All statistical analyses were conducted using SPSS for Windows (version 22, Chicago, IL, USA), and a *p*-value of < 0.05 was considered significant.

## 3. Results

The data of 126 patients with ARDS who received lung biopsies after 2003 were collected; of these patients, 68 had DAD, 51 did not have DAD, and 7 had IPF. Among the 68 patients with DAD, we excluded 21 cases with a time from HRCT to ARDS diagnosis of more than 7 days, 6 cases with no available HRCT, and 5 patients with chronic lung disease to avoid false increases in the HRCT score. The remaining 34 patients with a time from HRCT to ARDS diagnosis of <7 days were enrolled (Figure 1), with 17 categorized in the 60-day survival group and 17 in the 60-day nonsurvival group. The main clinical characteristics of these 34 cases are summarized in Table 1. No significant differences were noted in the clinical parameters except the HRCT score between the survival and nonsurvival groups.

The HRCT abnormalities and HRCT scores are listed in Table 2. No significant differences in any of the six HRCT findings were observed between the survival and nonsurvival groups. However, the mean HRCT score and the mean total percentage of the area with traction bronchiectasis or bronchiolectasis were significantly higher in the nonsurvival group (272.7 ± 49.4; 34.0 ± 16.6) than in the survival group (241.9 ± 47.2; 19.6 ± 17.3), indicating more fibroproliferative change in the nonsurvival group than in the survival group at an early stage of diagnosis.

The binary logistic regression model was applied to investigate whether the severity of illness (SOFA score), the severity of ARDS (PaO_2_/FiO_2_ ratio), impairment of pulmonary mechanics (dynamic driving pressure and dynamic MP), and the severity of parenchyma HRCT abnormality (HRCT score and total percentage of area with traction bronchiectasis) were independently associated with 60-day mortality. The dynamic MP (odds ratio (OR), 1.325; 95% CI, 1.018–1.723) and the total percentage of the area with traction bronchiectasis or bronchiolectasis (OR, 1.174; 95% CI, 1.011–1.326) positively predicted 60-day mortality (Table 3). Both these variables were input into the multiple logistic regression model, and only the total percentage of the area with traction bronchiectasis or bronchiolectasis remained an independent positive predictor of 60-day mortality (OR, 1.067; 95% CI, 1.011–1.126).

**Figure 3:**
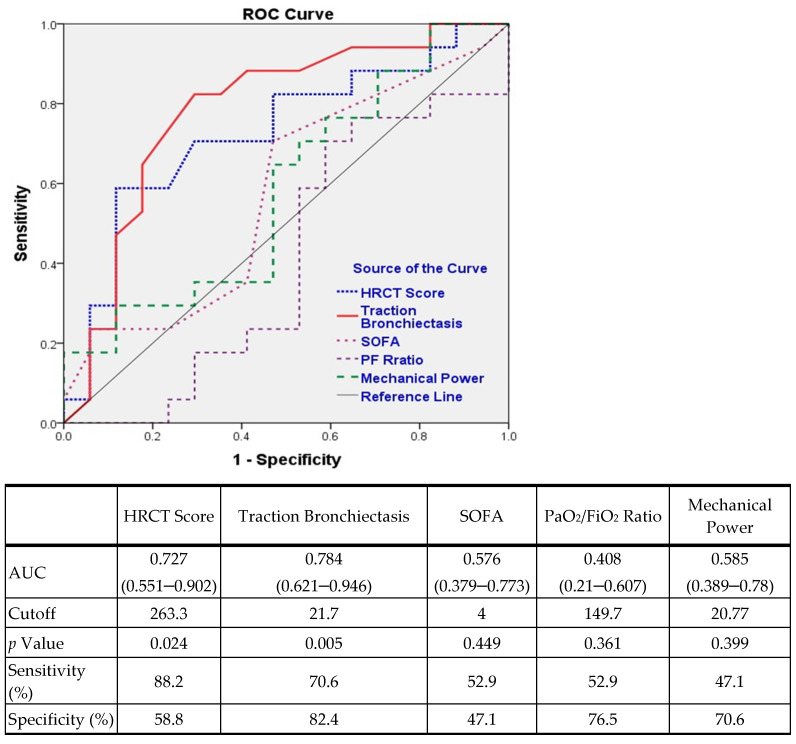
Receiver operator characteristic (ROC) curve of the HRCT score, traction bronchiectasis, SOFA score, PaO_2_/FiO_2_ ratio, and MP.

The ROC curve and the corresponding AUC were used to assess the performance of the aforementioned severity variables in predicting 60-day mortality and to determine the cutoff values of the statistically significant variables (Figure 3). The total percentage of the area with traction bronchiectasis or bronchiolectasis had a favorable predictive value [22] and the highest AUC (AUC, 0.784; cutoff, 21.7) among the five tested severity variables. The AUC for the HRCT score was 0.727, with a cutoff value of 263.3. The predictive abilities of the SOFA score, P/F ratio, and dynamic MP were no better than chance.

The results of the linear regression and correlation analysis revealed no significant correlation between the HRCT score and the dynamic MP (*p* = 0.29; R = −0.18) or P/F ratio (*p* = 0.927; R = 0.163) or between the total percentage of the area with traction bronchiectasis and the MP (*p* = 0.148; R = −0.25) or P/F ratio (*p* = 0.36; R = 0.16).

## 4. Discussion

The main study findings are as follows. (1) The HRCT score and the total percentage of the area with traction bronchiectasis or bronchiolectasis were significantly higher in the 60-day nonsurvival group than in the survival group. (2) As an alternative to the HRCT score, the total percentage of the area with traction bronchiectasis or bronchiolectasis was an independent predictor of 60-day mortality and exhibited a favorable predictive value (AUC, 0.784; 95% CI, 0.621–0.946). (3) Physiologic variables, including age, days from ARDS to HRCT, SOFA score, P/F ratio, dynamic driving pressure, and dynamic MP, were not discriminative between the 60-day survival and nonsurvival groups. (4) The HRCT findings, including both the HRCT score and the total percentage of the area with traction bronchiectasis or bronchiolectasis, were not correlated with ARDS severity (P/F ratio) or the impairment of pulmonary mechanics (dynamic MP).

DAD has been proposed to be the histological hallmark of acute-phase ARDS [3]. However, recent OLB [6,7,8] and autopsy [5,23,24,25] studies have consistently reported that only approximately half of ARDS cases diagnosed on the basis of the Berlin definition had DAD. Both the meta-analysis (*n* = 350) [6] and the PREDATOR study (*n* = 258) [9] by Cardinal-Fernandez et al. similarly demonstrated that those with DAD had a poorer prognosis than those without DAD, with the risk of hospital mortality approximately two times higher for those with DAD than that for those without DAD. Most of the aforementioned studies aimed to investigate the differences between cases with DAD and those without DAD and determine which of the physiological variables (e.g., SOFA score and P/F ratio) might be independently associated with DAD-related mortality. However, the results were inconsistent because of the differences in the enrolled patient population and sample size among the studies. Moreover, a model for distinguishing cases with DAD from those without DAD could not be established [6,9]. The PREDATOR [9] study concluded that histological DAD could not be predicted on the basis of clinical variables. By contrast, our study focused on the prediction of pathologically confirmed DAD. However, we found no significant difference in physiological variables between the survival and nonsurvival groups (Table 1).

The acute exudative phase of DAD occurs mainly in the first week after ARDS onset, followed by the fibroproliferative and fibrotic phases from the second week onward [4]. However, with the heterogeneity and different progression speeds of the disease, the time from the onset of the disease to progression to severity levels adequate to meet the ARDS Berlin definition criteria (e.g., P/F ratio < 300 mmHg and PEEP > 5 cmH_2_O) is variable and unpredictable. In a study of 159 autopsies of cases of ARDS with DAD, Thille et al. [26] reported that the prevalence of exudative change was 90% in 82 cases with an ARDS duration of less than 1 week. However, up to 54% (44/82) of the cases already exhibited fibroproliferative change, and 4% (3/82) had fibrosis.

Regarding HRCT manifestations, the study series of Ichikado et al. [14,15,16] demonstrated an association between the presence of traction bronchiectasis or bronchiolectasis in areas of GGO or consolidation and the late fibroproliferative or fibrotic phases. Fibroproliferation with traction bronchiectasis was observed in 64% (28/44) of patients who received HRCT within 7 days of ARDS diagnosis [17] and in 47% (40/85) of patients who received HRCT on the day of ARDS diagnosis [18]. These results emphasize that a clinically early phase of ARDS does not correspond to a pathologically early phase. However, through the HRCT changes, we could clearly estimate the pathological stage the patient was in at the time of ARDS diagnosis.

Lamy et al. [27] applied pathological findings to predict the prognosis of 45 OLB- or autopsy-confirmed ARDS cases and demonstrated that patients who exhibited histological acute exudative changes had a more favorable prognosis than patients in the fibroproliferative or fibrotic stages. Progression from the early exudative phase to the late fibroproliferative and fibrotic stages led to an impairment of lung mechanics and oxygen diffusion capacity, resulting in patients with ARDS being dependent on ventilators and susceptible to subsequent ventilator-associated pneumonia. Sepsis and multiple system organ failure during long-term ICU stays have been suggested to be the main causes of death in patients with ARDS [28,29], which may partly account for those with DAD having a poorer prognosis than those without DAD.

In the Ichikado HRCT scoring system, the score for traction bronchiectasis is weighted by multiplying the percent area of GGO with traction bronchiectasis by 4 and multiplying the percent area of consolidation with traction bronchiectasis by 5. Ichikado et al. [15,18] concluded that the HRCT score for pulmonary fibroproliferation assessment was the only independent predictor of susceptibility to multiple organ failure and ARDS outcomes. However, they did not test the original unweighted total percentage of area with traction bronchiectasis or bronchiolectasis. In our study, the Ichikado score of HRCT performed within 7 days of ARDS diagnosis failed to predict the outcomes, and only the total percentage of the area with traction bronchiectasis or bronchiolectasis was independently associated with 60-day mortality in patients with pathologically confirmed DAD. Ichikado et al. enrolled all relevant patients with ARDS in their cohort, whereas we only focused on ARDS patients with DAD. This difference in the enrolled populations might have contributed to the difference in the predictive values between the Ichikado HRCT score and the unweighted total percentage of the area with traction bronchiectasis. Nonetheless, our results confirmed the findings of Lamy et al. [27] and Ichikado et al. [15] showing that progression into the fibroproliferative or fibrotic stages early at the time of ARDS diagnosis was an independent predictor of poor prognosis.

In the present study, the ROC curve revealed that the values of the parameters of the severity of illness (SOFA score), the severity of ARDS (P/F ratio), and lung mechanics (dynamic MP) in the prediction of 60-day mortality related to DAD were not better than chance. Only the HRCT findings significantly predicted 60-day mortality. The total percentage of the area with traction bronchiectasis demonstrated a favorable predictive value. with the highest AUC of 0.784 (95% CI, 0.621–0.946; sensitivity, 70.6%; specificity, 82.4%) and a cutoff of 21.7. The mortality for the subgroup with a total percentage of the area with traction bronchiectasis of >21.7 was 74% (14/19), and that for the subgroup with a value of ≤21.7% was 20% (3/15). Roughly, in patients with DAD with a total area of fibroproliferation of >22% at the time of ARDS diagnosis, the mortality rate was approximately 3.7 times higher than that in patients with <22% fibroproliferation. The AUC for the HRCT score was 0.727 (95% CI, 0.551–0.902; sensitivity, 88.2%; specificity, 58.5%), and the cutoff was 263.3. This was higher than the cutoff of 210 in Ichikado’s study [18], which may imply that those with DAD have more severe fibroproliferation than those without DAD at the time of ARDS diagnosis. Notably, the AUC of the HRCT score for predicting 60-day survival in Ichikado’s whole ARDS cohort (0.71) was similar to the AUC of the HRCT score in our DAD cohort (0.72). Our results highlight the consistency of the Ichikado HRCT scoring system in predicting ARDS survival and underscore the superior predictive value of the total percentage of the area with traction bronchiectasis or bronchiolectasis.

Amato et al. proposed that driving pressure was the factor most associated with mortality in ARDS and that decreasing the driving pressure through the adjustment of ventilatory settings was strongly associated with increased survival [30]. The driving pressure of the respiratory system is defined as the ratio of the tidal volume to respiratory system compliance or the difference between the plateau pressure and PEEP. A meta-analysis [31] comprising 3252 patients also confirmed the association between high driving pressure and high mortality (OR, 1.44; 95% CI, 0.11–1.18). Thille et al. [25], in their study of 159 autopsies of cases of ARDS with DAD, also demonstrated that increased dynamic driving pressure was independently associated with DAD. In addition to driving pressure, MP incorporates the tidal volume, pressure, flow, and respiratory rate to calculate the amount of energy delivered to the respiratory system by the ventilator per unit of time. MP is superior to other ventilator parameters in estimating the risk of ventilator-associated lung injury [32] and can enable risk estimation using factors other than driving pressure alone. MP has been shown to predict mortality in critically ill [33] and ARDS patients [34,35]. A study that applied dynamic variables and a registry-based prospective cohort containing 13,939 patients, including those with ARDS, also revealed that increases in the dynamic driving pressure and dynamic MP were associated with an increased risk of ICU mortality [21]. On the contrary, the dynamic driving pressure was correlated with neither DAD nor mortality in the PREDATOR study [9] and was also not associated with mortality related to ARDS with DAD in our cohort (Table 3). In our study, although MP was significant in the univariate regression analysis, the multivariate logistic regression analysis failed to confirm that dynamic MP was an independent predictor of 60-day mortality related to DAD (Table 3).

Our study has some limitations. First, the results may have been influenced by selection bias. OLB was performed only for ARDS patients presenting bilateral GGO or consolidation with rapid progression and who did not exhibit clear etiology of ARDS. Therefore, AIP cases accounted for a large proportion of our cohort. However, Parambil et al. [36] reported that infections and AIP were the most common causes of DAD diagnosed using OLB. Therefore, our results might still be applicable to DAD with other underlying etiologies. Second, this study is retrospective in nature and has a relatively small sample size because only a small proportion of ARDS patients were selected for OLB. Furthermore, the patients were required to have received HRCT within 7 days of ARDS diagnosis to assess the severity of fibroproliferation. Approximately 40% (27/68) of the pathologically confirmed DAD cases in our cohort were excluded because of the lack of HRCT within 7 days of ARDS diagnosis (Figure 1). The results might have been more conclusive if these 27 cases were enrolled.

## 5. Conclusions

As an alternative to the HRCT score, the extent of fibroproliferation on HRCT performed within 7 days of ARDS diagnosis, denoted as the total percentage of the area with traction bronchiectasis or bronchiolectasis, was an independent predictor and had a favorable predictive value for the 60-day mortality of patients with pathologically confirmed ARDS with DAD. A total fibroproliferation percentage greater than the cutoff of 22% signified poor prognosis and a 3.7 times higher 60-day mortality. Physiological variables could not adequately discriminate between survival and nonsurvival cases.

## Figures and Tables

**Table 1:** Clinical Characteristics of ARDS Patients with DAD.

Charteristics	Total (*n* = 34)	Survivors (*n* = 17)	Non-Survivors (*n* = 17)	*p* Value
Age (years)	58.9 ± 16	56.5 ± 13.8	61.4 ± 18	0.255
Sex (male/female)	20/14	9/8	11/6	0.21
**Cause of ARDS**				
AIP	17	7	10	
Pneumonia	9	3	6	
Sepsis induced ARDS	3	2	1	
Methotrexate induced DAD	2	2		
Autoimmune interstitial lung disease	1	1		
Amphetamine induced ARDS	1	1		
Cytomegalovirus pneumonitis	1	1		
**Severity of ARDS**				
mild	26.5% (9/34)	35.3% (6/17)	17.6% (3/17)	
moderate	61.8% (2/34)	52.9% (9/17)	64.7% (11/17)	
severe	14.7% (5/34)	11.8% (2/17)	17.6% (3/17)	
Days from ARDS to HRCT (days)	1.9 ± 1.8	1.9 ± 2.0	1.9 ± 1.6	0.206
Days from ARDS to lung biopsy (days)	8.5 ± 8.7	9.0 ± 11.6	7.1 ± 4.5	0.308
Days from HRCT to lung biopsy (days)	7.2 ± 8.9	9.9± 11.4	5.4 ± 4.8	0.467
Duration of mechanical duration (days)	29.9 ± 34.1	35 ± 46.8	24.8 ± 12.3	0.563
SOFA score	5.2 ± 2.2	4.9 ± 1.9	5.6 ± 2.4	0.443
HRCT score	257.3 ± 49.3	241.9 ± 47.2	272.7 ± 51.9 *	0.024
PaO_2_/FiO_2_	154.8 ± 61.1	169.1 ± 69.5	140.5 ± 49.2	0.361
**Ventilator variables**				
Tidal Volume (mL/kg predicted)	7.7 ± 2.3	7.4 ± 2.2	8.0 ± 2.4	0.836
PEEP (cmH_2_O)	11.9 ± 2.6	11.8 ± 2.6	12.1 ± 2.6	0.644
Dynamic driving pressure (cmH_2_O)	19.6 ± 5.0	20.7 ± 5.1	18.5 ± 4.8	0.161
Mechanical Power (J/min)	22.7 ± 5.1	22.1 ± 5.2	23.4 ± 5.1	0.391

**Abbreviation:** AIP: acute interstitial pneumonia; DAD: diffuse alveolar damage; SOFA: sequential organ failure assessment; PEEP positive end-expiratory pressure. * *p* value < 0.05 between survival and nonsurvival.

**Table 2:** HRCT score and HRCT findings of survivors and non-survivors.

	All Patients (*n* = 34)	Survivals (*n* = 17)	Non-Survivals (*n* = 17)	*p* Value
HRCT score	257.3 ± 49.3	241.9 ± 47.2	272.7 ± 49.4 *	0.024
Percentage of area without traction bronchiectasis	73.2 ± 18.2	80.4 ± 17.3	66.1 ± 16.6 *	0.005
Percentage of area with traction bronchiectasis	26.8 ± 18.2	19.6 ± 17.3	34.0 ± 16.6 *	0.005
Normal Attenuation	22.5 ± 18.2	20.7 ± 18.3	24.2 ± 18.5	0.523
Ground-Glass opacity	36.1 ± 20.7	43.1 ± 20.6	29.1 ± 18.9	0.108
Consolidation	14.7 ± 15.6	16.6 ± 19.6	12.7 ± 10.5	0.809
Ground-Glass opacity with traction bronchiectasis or bronchiolectasis	16.1 ± 12.9	12.8 ± 12.2	19.4 ± 13.1	0.097
Consolidation with traction bronchiectasis or bronchiolectasis	10 ± 9.6	6.8 ± 7.3	13.2 ± 10.8	0.054
Honeycombing	0.7 ± 2.9	0 ± 0	1.4 ± 4.1	0.074

* *p* value < 0.05 between survival and nonsurvival.

**Table 3:** Univariate and multivariate logistic regression for analyzing independent risk factors for 60-day mortality.

Variables	Univariate OR (95% CI)	*p* Value	Multivariate OR (95% CI)	*p* Value
SOFA	0.901 (0.506–1.602)	0.722		
PaO_2_/FiO_2_ ratio	1.018 (0.999–1.037)	0.069	0.984 (0.969–1.000)	0.054
Driving pressure	1.275 (0.96–1.694)	0.093		
Mechanical Power	0.755 (0.58–0.982)	0.036	1.162 (0.971–1.391)	0.102
HRCT score	1.038 (0.985–1.093)	0.162		
% of total area with traction bronchiectasis or bronchiolectasis	0.852 (0.734–0.989)	0.035	1.082 (1.021–1.148)	0.008

**Abbreviations:** OR: odds ratio; CI: confidence interval.

## Data Availability

The data sets analyzed in the study are available from the corresponding author upon reasonable request.
